# Data on genome assembly and annotation of *Marinobacter* sp. strain CA1 isolated from indigenous diatom found in whiteleg shrimp pond in Malaysia

**DOI:** 10.1016/j.dib.2022.108049

**Published:** 2022-03-11

**Authors:** Sarmila Muthukrishnan, Hirzahida Mohd-Padil, Norfarrah Mohamed-Alipiah, Ikhsan Natrah

**Affiliations:** a Department of Aquaculture, Faculty of Agriculture, Universiti Putra Malaysia, Serdang, Selangor 43400, Malaysia; b Aquatic Animal Health and Therapeutics Laboratory, Institute of Bioscience, Universiti Putra Malaysia, UPM, Serdang, Selangor 43400, Malaysia

**Keywords:** *Marinobacter*, Illumina sequencing, Pacbio sequencing, Genome assembly, NGS sequencing

## Abstract

*Marinobacter* is a genus belonging to the class Gammaproteobacteria and the family Alteromonadaceae. This genus is a Gram-negative bacterium which can be found in a wide range of marine and saline water environments. Here, we present the genome sequence of *Marinobacter* sp. strain CA1 that was isolated from the indigenous diatom found in the whiteleg shrimp, *Penaeus vannamei* (Bonne 1931) pond in Malaysia. Genome sequencing was done using Pacbio and Illumina sequencing platforms. *De novo* hybrid assembly based on Pacbio long reads and high quality Illumina short reads yielded a complete circular chromosome with 4.7 M base pair (bp) in size. This genome was submitted to Genbank, NCBI database and can be accessed under accession number: NZ_CP071785 and the BioProject acession number PRJNA710741. This data could be useful for other studies on microalgae and bacteria interaction, and comparative genomics analysis of other *Marinobacter* species.

## Specifications Table

**Table:** 

Subject	Molecular biology
Specific subject area	Microbiology and Genomics
Type of data	Complete genome assembly in FASTA format Figure Table
How data were acquired	Genomic data was extracted and whole genome sequencing was done using Pacbio and Illumina sequencing platforms. Raw reads were assembled and genome was annotated using variety of bioinformatics tools.
Data format	Analysed data in FASTA format
Parameters for data collection	Genomic DNA was extracted from the culture of *Marinobacter* sp. strain CA1 isolated from a diatom found in a Malaysian shrimp pond.
Description of data collection	Sequencing reads were generated on the Illumina and PacBio platforms. Raw sequencing reads were subjected to quality control and adapter trimming. Hybrid assembly of reads from both platforms then performed using Unicycler version v0.4.9b. Genome was annotated using Prokaryotic Genome Annotation Pipeline (PGAP) pipeline in NCBI.
Data source location	City/Town/Region: Terengganu Coordinate: 5°34′18.3″ N and 102°48′29.2″ E Country: Malaysia
Data accessibility	The data is hosted on a public repository. Repository name: NCBI database Accession number: NZ_CP071785 Direct URL: https://www.ncbi.nlm.nih.gov/nuccore/NZ_CP071785

## Value of the Data

• The data of *Marinobacter* sp. is useful to describe its genomic variation and would contribute to the understanding of its potential as a quorum quencher.
• The genome data deposited to a public biological database may benefit researchers working on genomics analysis of bacteria from marine environments, specifically those associated with microalgae.
• The data of *Marinobacter* sp. can be used for comparative genomic analysis useful to be included in comparative genomic analysis to understand the evolution of this bacterial species, genetic adaptation through mutation or horizontal gene transfer, and possible biotechnology application based on its genomic content.

## 1 Data Description

We present here the complete genome assembly of *Marinobacter* sp. strain CA1 that is associated with a diatom species isolated from the whiteleg shrimp, *Penaeus vannamei* pond in Malaysia. The whole genome sequencing of Marinobacter sp. CA1 generated 4,648,785 paired-end reads and 45,563 reads from Illumina and PacBio platforms, respectively. The genome *Marinobacter* sp. strain CA1 was sequenced to 100 × depth using Illumina and 50 × depth using Pacbio platform. *De novo* hybrid assembly of high-quality Illumina short reads and Pacbio long reads yielded a complete circular chromosome with 4,721,802 bp in length and a GC content of 60.99%. The assembly coverage exhibited 47× which was calculated based on overall 225,261,560 bases that mapped to the genome. A total of 12 ribosomal RNAs (rRNAs), 57 transfer RNAs (tRNAs) and 4297 genes were predicted and annotated using Prokaryotic Genome Annotation Pipeline (PGAP) pipeline in National Center for Biotechnology Information (NCBI).

**Fig. 1:**
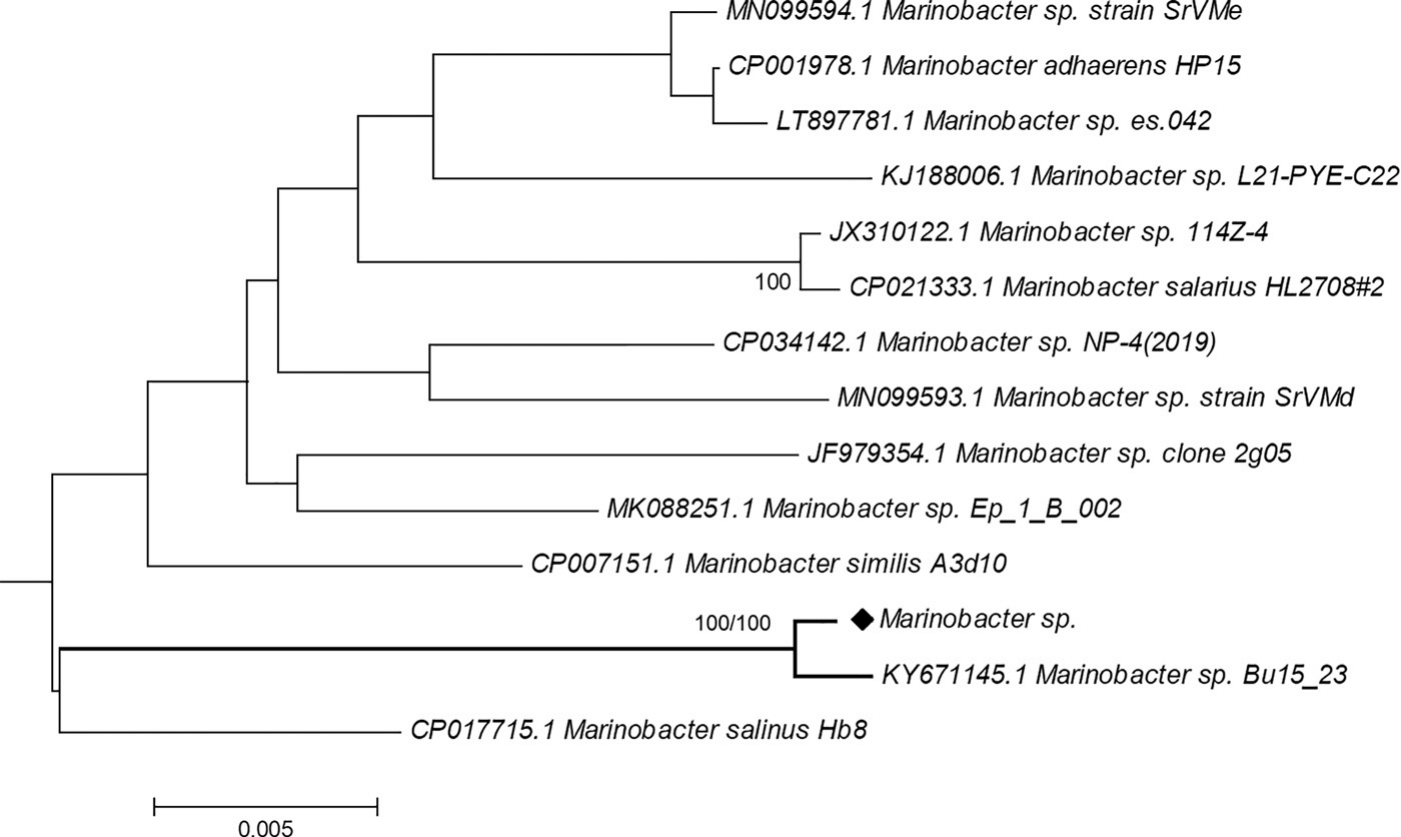
Phylogenetic trees of 16S rRNA sequences of *Marinobacter* bacteria. As the topology of both Neighbor-Joining (NJ) and Maximum-Likelihood (ML) are almost similar, only a consensus tree is shown here with bootstrap value represented as (NJ/ML). In both phylogenetic trees with 1000 bootstrap replicates, 16S rRNA sequence from *Marinobacter* sp. strain CA1 (marked with a diamond symbol) was clustered together with *Marinobacter* sp. BU15_23 with 100% bootstrap value.

Based on the 16S rRNA sequence phylogenetic tree of this isolate and other 13 homologous 16S rRNA sequences, this bacteria was found to be closely related with *Marinobacter* sp. Bu15_23 supported with 100% bootstrap values in both Neighbour-Joining and Maximum-Likelihood trees (Fig. 1) and the data made available in Newick format in Supplementary data. Two betalactone, 1 redox-cofactor and 1 ectoine clusters were identified under biosynthetic gene clusters (BGCs) using AntiSMASH. The betalactone cluster identified from nucleotide 826,411 to 853,236 is 20% homologous to known cluster that produces fengycin secondary metabolite. Redox-cofactor cluster located from nucleotide 3,864,498 to 3,886,676 is 13% similar to the known cluster that produces lankacidin C secondary metabolite (Table 1).

**Table 1:** Biosynthetic genes clusters identified in Marinobacter sp. CA1 based on AntiSMASH screening.

Type	Start	Stop	Most similar know cluster	Similarity
Betalactone	826,411	853,236	Fengycin	20%
Betalactone	1977,298	2003,105		
Redox-cofactor	3864,498	3886,676	Lankacidin C	13%
Ectoine	4460,973	4471,359		

## 2 Experimental Design, Materials and Methods

### 2.1 Sample preparation

The diatom sample was isolated from a shrimp farm located at the East Coast of Peninsular Malaysia. A total of 100 μL suspensions was inoculated into 10 mL Marine Broth (MB) (Difco™, Detroit, USA). The inoculum was incubated at 28 °C overnight under constant agitation. Then, 100 μL of the sample from the overnight grown culture was plated on Marine Agar (MA) (Difco™, Detroit, USA) using spread plate technique, incubated at 28 °C overnight before the single colony was inoculated into 10 mL of MB. The inoculum was incubated at 28 °C overnight under constant agitation. The pure inoculum was centrifuged at 4500 *g* for 10 min and the supernatants were discarded. The cell-pellets (in triplicates) were submerged in 10 mL of molecular grade ethanol (SYSTERM, Malaysia) and submitted to Apical Scientific Sdn. Bhd. (Selangor, Seri Kembangan, Malaysia) for sequencing.

### 2.2 Genome sequencing and assembly

Genome sequencing was done using Pacbio and Illumina sequencing platforms. For Illumina paired end data, the pre-processing step to remove sequence adaptors, reads with length shorter than 50 bp, reads with quality score lower than Qv = 20 was carried out using SolexaQA++ v*.*3.1.7.2 tool [1]. *De novo* hybrid assembly of PacBio long reads and high quality short reads from Illumina was done using Unicycler v.0.4.9.b program [2].

### 2.3 Gene prediction and annotation

The genome was submitted to Genbank, NCBI database. Genome annotation was done using Prokaryotic Genome Annotation Pipeline (PGAP) in NCBI [3]. Using this pipeline, tRNA and rRNA sequences were predicted using tRNAscan-SE and BLASTN against the rRNA reference set, respectively. GeneMarkS+ was used to predict the protein-coding before annotated using BLASTP search against protein databases. The protein sequences were named based on similar rules adopted by the Uniprot-SwissProt Protein Knowledgebase [4].

### 2.4 Comparative genome analysis

For phylogenetic analysis, nucleotide sequences of other bacteria that are homologous to 16S rRNA sequence of the bacteria were searched using BLASTN program against NCBI 16S rRNA database. The sequences then were aligned using MUSCLE v3.8.3.1 software [5] before the output alignment was used as an input to construct Neighbor-Joining and Maximum-Likelihood phylogenetic trees using phylogeny.fr [6] and PhyML 3.0 [7], respectively, with 1000 bootstrap samples. Search of gene clusters for secondary metabolite production was conducted using the antiSMASH 5.0 program [8].

## Ethics Statement

This work did not involve the use of human subjects or animal experiments.

## CRediT authorship contribution statement

**Sarmila Muthukrishnan:** Conceptualization, Methodology, Writing – original draft. **Hirzahida Mohd-Padil:** Methodology, Software, Data curation, Writing – original draft. **Norfarrah Mohamed-Alipiah:** Writing – original draft. **Ikhsan Natrah:** Supervision, Writing – review & editing.

## Declaration of Competing Interest

The authors declare that they have no known competing financial interests or personal relationships which could be perceived to influence the work reported in this article.

## Appendix Supplementary materials

Image, application 1

## References

[1] Cox M.P., Peterson D.A., Biggs P.J. (2010). SolexaQA: at-a-glance quality assessment of Illumina second-generation sequencing data. BMC Bioinform..

[2] Wick R.R., Judd L.M., Gorrie C.L., Holt K.E. (2017). Unicycler: resolving bacterial genome assemblies from short and long sequencing reads. PLoS Comput. Biol..

[3] Tatusova T., DiCuccio M., Badretdin A., Chetvernin V., Nawrocki E.P., Zaslavsky L., Lomsadze A., Pruitt K.D., Borodovsky M., Ostell J. (2016). NCBI prokaryotic genome annotation pipeline. Nucleic Acids Res..

[4] Altschul S.F., Gish W., Miller W., Myers E.W., Lipman D.J. (1990). Basic local alignment search tool. J. Mol. Biol..

[5] Edgar R.C. (2004). MUSCLE: multiple sequence alignment with high accuracy and high throughput. Nucleic Acids Res..

[6] Dereeper A., Guignon V., Blanc G., Audic S., Buffet S., Chevenet F., Dufayard J.F., Guindon S., Lefort V., Lescot M., Claverie J.M., Gascuel O. (2008). Phylogeny.fr: robust phylogenetic analysis for the non-specialist. Nucleic Acids Res..

[7] Guindon S., Dufayard J.F., Lefort V., Anisimova M., Hordijk W., Gascuel O. (2010). New algorithms and methods to estimate maximum-likelihood phylogenies: assessing the performance of PhyML 3.0. Syst. Biol..

[8] Blin K., Shaw S., Steinke K., Villebro R., Ziemert N., Lee S.Y., Medema M.H., Weber T. (2019). antiSMASH 5.0: updates to the secondary metabolite genome mining pipeline. Nucleic Acids Res..

