# Supplementary material for: Data on genome assembly and annotation of Marinobacter sp. strain CA1 isolated from indigenous diatom found in whiteleg shrimp pond in Malaysia
Source: Data Brief. 2022 Mar 11;42:108049. doi: 10.1016/j.dib.2022.108049 (PMC8943393; doi:10.1016/j.dib.2022.108049)
Supplement: Supplementary file 1 [file mmc1.docx]

((CP007151.1_Marinobacter_similis_A3d10:0.00839000,((JF979354.1_Marinobacter_sp._clone_2g05:0.01126000,MK088251.1_Marinobacter_sp._EP_1_B_002:0.00675000)0.4000:0.00112000,((((MN099594.1_Marinobacter_sp._strain_SrVMe:0.00102000,(CP001978.1_Marinobacter_adhaerens_HP15:0.00012000,LT897781.1_Marinobacter_sp.042:0.00120000)0.9400:0.00096000)1.0000:0.00533000,KJ188006.1_Marinobacter_sp._L21-PYE-C22:0.00984000)0.4800:0.00169000,(JX310122.1_Marinobacter_sp._114Z-4:0.00044000,CP021333.1_Marinobacter_salarius_HL2708_2:0.00087000)1.0000:0.00994000)0.5400:0.00180000,('CP034142.1 Marinobacter sp. NP-4(2019)':0.00636000,MN099593.1_Marinobacter_sp._strain_SrVMd:0.00894000)0.7200:0.00341000)0.2200:0.00069000)0.4500:0.00223000)0.5900:0.00216000,((Marinobacter_sp.:0.00091000,KY671145.1_Marinobacter_sp._Bu15_23:0.00172000)1.0000:0.01652000,CP017715.1_Marinobacter_salinus_Hb8:0.00766000)0.5900:0.00017000);
